# Endo-PairGS: pair priors for dynamic endoscopic scene reconstruction

**DOI:** 10.1007/s11548-026-03707-y

**Published:** 2026-05-21

**Authors:** Xiankang Yu, Yuichiro Hayashi, Masahiro Oda, Takayuki Kitasaka, Kensaku Mori

**Affiliations:** 1 Graduate School of Informatics, Nagoya University, Furo-cho, Chikusaku, Nagoya, Aichi Japan; 2 Information Technology Center, Nagoya University, Furo-cho, Chikusaku, Nagoya, Aichi Japan; 3 School of Information Science, Aichi Institute of Technology, 1247 Yachigusa, Yagasa-cho, Toyota, Aichi Japan; 4 Research Center for Medical Bigdata, National Institute of Informatics, 2-1-2 Hitotsubashi, Chiyoda-ku, Tokyo, Japan

**Keywords:** 3D reconstruction, 4D Gaussian splatting, Dynamic endoscopic scene, Foundation model fine-tuning

## Abstract

**Purpose:**

Dynamic scene reconstruction in endoscopic data is crucial for minimally invasive surgery and surgical navigation. Recently, Gaussian splatting (GS)-based methods have performed outstandingly in the reconstruction of dynamic endoscopic scenes. However, more challenging scenes with deformable tissues, surgical tool occlusion, and changeable camera positions make it harder to initialize a GS model well. To address this, we propose Endo-PairGS, which leverages aligned point cloud pairs from two different frames to initialize a 4D GS model.

**Methods:**

Our framework contains two parts: static and dynamic 3D reconstruction. Firstly, we fine-tune a foundation model on endoscopic data to obtain aligned point clouds. To ensure the stability of fine-tuning video sequences with deformable tissues, we use an optical flow-based mask to reduce the influence of regions with large movement. Then, we used the generated point cloud pairs to initialize a 4D GS model for dynamic scene reconstruction. The paired point clouds can complement the region masked by the surgical tool and the unseen region caused by the camera position change, which presents a more complete 3D scene.

**Results:**

The experiments on EndoNeRF and StereoMIS datasets demonstrate our Endo-PairGS outperformed existing methods on both quantitative and qualitative results. Our method got 32.47 and 0.871 on PSNR and SSIM in StereoMIS (P3), improving 4% and 4.8% compared to the baseline approach, respectively.

**Conclusion:**

This study proposed Endo-PairGS with a more complete initialization method, significantly improving the performance of dynamic endoscopic scene reconstruction. The codes and data will be released at https://github.com/MoriLabNU/Endo-PairGS.

## Introduction

Dynamic scenes mean that objects in a video sequence will move along the timeline, such as actions or position changes. In endoscopic scenes, the situation will be more complicated with limited field-of-view, deformable tissues, surgical tool occlusion, and changeable camera positions. For endoscopic surgical procedures, extracting dynamic 3D information from monocular video sequences is critical to navigate complex surgery, especially for minimally invasive surgery. Although it is challenging to reconstruct the dynamic endoscopic scene, accurate dynamic 3D environments will enhance surgeons’ spatial understanding and navigation.

Recently, deep learning-based methods for 3D reconstruction in endoscopy showed significant improvement compared to traditional approaches, such as structure from motion (SfM) [1] and multiview stereo (MVS) [2]. Neural radiance fields (NeRFs) [3] and Gaussian splatting (GS) [4] are two popular learnable methods used in endoscopic scene reconstruction. For the dynamic scene reconstruction, recent works proposed 4D GS, which uses an extra time dimension to model the movements between frames. EndoNeRF [5] leveraged a dynamic NeRF-based method with MLPs to optimize stereo 3D reconstruction of deformable tissues. EndoSurf [6] adopted three neural fields to present surface dynamics, shape, and texture with the aim of improving the quality of geometry and appearance. EndoSelf [7] utilized a self-supervised 3D reconstruction method with the geometric constraint of depth values and the looped optimization for temporal information. To accelerate training and inference, Endo-4DGS [8] presents a 4D Gaussian splatting method with pseudo-depths as initialization prior and guide label. EndoGS [9] combined static Gaussians and deformable parameters in the time dimension, associated with depth supervision, to achieve a faster 3D reconstruction. Deform3DGS [10] utilized motion-aware point fusion to initialize the dense Gaussian point cloud and flexible deformation modeling to represent tissues.

Although NeRF-based methods [5–7] have exhibited powerful 3D reconstruction performance, their implicit representations have limited training speed and downstream practical applications. The explicit methods, like GS [8–14] and ForPlane [15], have demonstrated advantages in low computation cost and fine surface reconstruction. However, a superior initialization method will impact the final performance of the GS-based approach. Recent GS-based methods used depth [8, 10–14] or SfM point clouds [9] to initialize the 3D Gaussians, but most endoscopic datasets do not have ground-truth point clouds or depths. Pseudo-depth emerged in many initialization approaches, like Endo-4DGS, which leveraged Depth Anything [16] to generate pseudo-depth for initialization. Although pseudo-depth can provide 3D information, the lack of scale and camera information makes it challenging to initialize a 3D scene well. Instead, pseudo-point clouds include both depth and camera information, which represents more accurate 3D environments. With the benefit of advanced 3D matching and reconstruction techniques [17–19], it is possible to acquire accurate 3D point clouds or even align 3D point cloud pairs to initialize a more complete 3D environment.

**Fig. 1:**
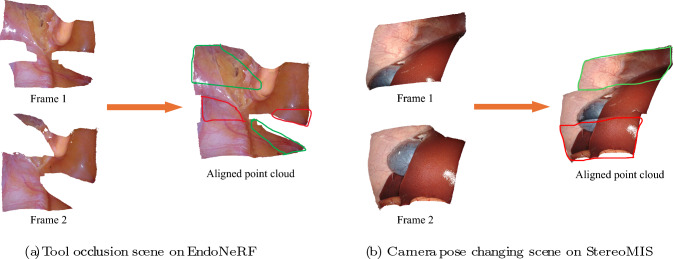
Examples of aligned point clouds on EndoNeRF [5] and StereoMIS [20] datasets. The red and green circles show the region missing in frames 1 and 2, respectively

Our framework, Endo-PairGS, proposes a solution to obtain pair- or multi-aligned 3D point clouds for initializing 3D Gaussians. Inspired by Splatt3R [19], we fine-tuned it on collected dynamic endoscopic scenes from the EndoNeRF [5] and StereoMIS [20] datasets. As Splatt3R is proposed for rigid bodies, it can determine a fixed match of corresponding points between input image pairs and target images. However, the tissues will deform in a video sequence that makes it harder to define a fixed match between input and target. To keep a stable fine-tuning procedure, an optical flow-based mask is proposed to reduce the impact of regions with significant changes. Firstly, we calculate the optical flow between two input frames to define the fixed region and then randomly mask the fixed region of generated pointmaps in a complementary manner to guarantee the efficient gradients of two pointmaps. We also leverage optical flow to judge the movement of pixels between input pairs and target frames, and then, we allocate a low weight to the pixel with a large optical flow value when calculating loss. For dynamic endoscopic scene reconstruction, we pick two frames with a large interval to generate an aligned point cloud pair for initializing an Endo-4DGS [8]. As shown in Fig. 1, paired point clouds will contain more extra information, which will be missing in a single frame due to tool occlusion or camera position change.

In summary, our main contributions are as follows: 1) We fine-tune the foundational 3D reconstruction method using an optical flow-based mask specifically for dynamic endoscopic scenes, which generates a well-aligned pair of point clouds from the endoscopic data. 2) The aligned point cloud pair is used for initializing 3D Gaussians and training a 4D Gaussian splatting model with depth guidance from the predicted 3D point cloud. 3) With comprehensive experiments on six dynamic endoscopic scenes extracted from the EndoNeRF [5] and StereoMIS [20] datasets, our methods outperformed other existing state-of-the-art (SOTA) methods in both quantitative results and rendering performance, especially the tissues with large tool occlusion.

## Method

**Fig. 2:**
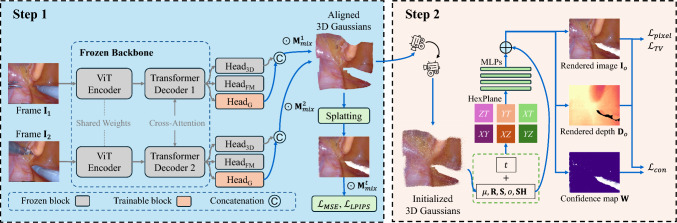
The overall architecture of our framework. **Step 1** is the fine-tuning procedure of the Gaussian head $\mathrm {Head_G}$ of Splatt3R [19]. **Step 2** leveraged the aligned point cloud to initialize the 3D Gaussians for dynamic scene reconstruction

To address the inferior initialization of 3D Gaussians in dynamic endoscopic scenes, this paper proposes a 2-step framework using a well-aligned 3D point cloud pair to obtain a larger tissue region than a single frame. In the first step, we fine-tune Splatt3R [19] on 20 endoscopic scenes collected from the EndoNeRF [5] and StereoMIS [20] datasets with the optical flow-based mask. Only the Splatt3R [19] output heads are fine-tuned to improve the accuracy of the aligned point cloud and estimated camera intrinsics. Secondly, we leverage Endo-4DGS [8] as the baseline model, initialized with an aligned point cloud pair from two frames with a large interval (over 20 frames) and trained with the pseudo-depth from point clouds generated by fine-tuned Splatt3R. The overall architecture of our framework is shown in Fig. 2.

### Aligning point clouds

In Fig. 1, it is evident that the aligned point cloud pair contains more tissue regions compared to the single frame in tool occlusion and camera pose changing situations. Previous point cloud registration methods [21], including iterative optimization-based, feature-based, and learning-based approaches, perform well with rigid objects but require point clouds as input. Unfortunately, most endoscopic data miss ground-truth point clouds or depths, making it difficult to directly use those methods.

With the help of the foundation models, Splatt3R [19] is utilized to generate aligned point clouds. As illustrated in Fig. 2, only paired frames are needed as input to generate aligned point maps in the camera coordinates of the first frame. Splatt3R creatively added a Gaussian head to represent the 3D structure and splatted it in the 2D plane for optimization. This training strategy is highly suitable for endoscopic data, for which only images can be used for guidance. However, Splatt3R was trained on rigid environments, and it may degrade on deformable tissues in the alignment of point clouds and prediction of the camera’s intrinsics. Due to the limited datasets and weak supervision, we fine-tuned the output Gaussian heads instead of the entire model to avoid the influence of pre-trained backbone parameters.

### Optical flow-based mask

An optical flow-based mask is proposed to improve fine-tuning stability in order to address the lack of 3D information and stationary camera in endoscopic data. In previous methods [17, 18] and Splatt3R [19], the content mask is needed for randomly selected input pairs and target images. They use the ground-truth 3D point cloud and the camera’s extrinsics to calculate the overlapping part between selected pairs to guarantee a suitable overlap rate of input. The overlapping region between input pairs and target images is calculated to ensure a valid loss during training. Nevertheless, most endoscopic data lack annotated 3D point clouds to calculate accurate overlap.

Instead, we use RAFT [22] to estimate an approximate overlapping and movement between frames. The pre-trained *RAFT* model is leveraged to calculate optical flow between input pairs ($\textbf{I}_1, \textbf{I}_2$) and the target frame $\textbf{I}_t$. The computation is shown as

1 $$\begin{aligned} \textbf{OF}_{1, 2}(i, j)&= \textbf{M}_{tool}^1(i,j) \textbf{M}_{tool}^2(i,j) \nonumber \\&\sqrt{RAFT_x^{\textbf{I}_1,\textbf{I}_2}(i,j)^2 +RAFT_y^{\textbf{I}_1,\textbf{I}_2}(i,j)^2}, \end{aligned}$$

where $\textbf{OF}_{1, 2}$ refers to the estimated optical flow magnitude of $\textbf{I}_1$ and $\textbf{I}_2$. $\textbf{M}_{tool}^1$ and $\textbf{M}_{tool}^2$ mean the surgical tool masks of $\textbf{I}_1$ and $\textbf{I}_2$. *i*, *j* is the size of $\textbf{I}_1$.

To ensure the gradient of both point clouds in a fixed camera situation, an overlapping mask $\textbf{M}_{overlap}$ is generated to randomly mask part of the overlapping region of input pairs complementarily. It allows rendered images to contain information from both point clouds even if they are 100% overlapped. The equations show as

2 $$\begin{aligned}&\textbf{M}_{overlap}^{1,2}(p, q)\nonumber \\&\quad = {\left\{ \begin{array}{ll} 0 &  \text {if }\textbf{OF}_{1, 2}(p, q)/MAX(\textbf{OF}_{1, 2}) \le \lambda _{thr}, \\ 1 &  \text {otherwise,} \end{array}\right. } \end{aligned}$$

3 $$\begin{aligned}&\textbf{M}_{overlap}^1(p,q)\nonumber \\&\quad = {\left\{ \begin{array}{ll} 0 &  (p,q)\in Rsample(\textbf{M}_{overlap}^{1,2})\ \text {and}\ \textbf{M}_{overlap}^{1,2}(p, q)=0,\\ 1 &  otherwise, \end{array}\right. } \end{aligned}$$

4 $$\begin{aligned}&\textbf{M}_{mix}^1 = \textbf{M}_{tool}^{1} \odot \textbf{M}_{overlap}^1 , \end{aligned}$$

where $\textbf{M}_{overlap}^{1,2}$ is the mask of the low-movement region between $\textbf{I}_1$ and $\textbf{I}_2$. *p*, *q* is the size of $\textbf{I}_1$. $\textbf{M}_{overlap}^1$ is a randomly sampled mask from the overlapping region, e.g., $\textbf{M}_{overlap}^2 = \textbf{1}-\textbf{M}_{overlap}^1+\textbf{M}_{overlap}^{1,2}$. $\textbf{M}_{mix}^1$ refers to the mixed mask of $\textbf{I}_1$, and $\textbf{1}$ is the all-one matrix with the shape of $\textbf{I}_1$. *Rsample* is a random sampling function, and $\lambda _{thr}$ is the threshold of normalized optical flow. ⊙ is element-wise multiplication. As the same, $\textbf{M}_{mix}^2 = \textbf{M}_{tool}^{2} \odot \textbf{M}_{overlap}^2$.

**Table 1:** Detailed information of six dynamic scenes for evaluation. Since only odd frames have surgical tool masks in the StereoMIS dataset, we used a frame interval of 2 in P2-5 and P3

	EndoNeRF [5]		StereoMIS [20]			
Attributes	Cutting	Pulling	P1	P2-2	P2-5	P3
Frame range	[1, 156]	[1, 63]	[802, 1001]	[1, 100]	[5301, 5499]	[9201, 9499]
Frame interval	1	1	1	1	2	2
Length	156	63	200	100	100	150
Tool included	✓	✓	–	–	✓	✓
Tool mask ratio	27.9%	33.8%	0	0	0.1%	10.2%

To reduce the impact of a large moving region when computing loss, $\textbf{M}_{move}$ is used to reassign the weight based on the movement of each pixel. The computational procedure is shown as

5 $$\begin{aligned} \textbf{M}_{move}^t&= \textbf{1} - NORM(MIN(\textbf{OF}_{1, t}, \textbf{OF}_{2, t})), \end{aligned}$$

6 $$\begin{aligned} \textbf{M}_{mix}^t&= \alpha (\textbf{M}_{tool}^{1}\vee \textbf{M}_{tool}^{2}) \odot \textbf{M}_{tool}^t \odot \textbf{M}_{move}^t, \end{aligned}$$

where NORM(·) denotes a normalization to (0, 1) by dividing the maximum and MIN(·) is an element-wise minimum. ∨ denotes the logical OR operation between $\textbf{M}_{tool}^{1}$, and $\textbf{M}_{tool}^{2}$. $\textbf{M}_{move}^t$ is the inverse value of normalized optical flow. $\textbf{M}_{mix}^t$ is the mixed mask of the target frame $\textbf{I}_t$, and α is the weight of $\textbf{M}_{mix}^t$.

### Dynamic scene reconstruction

3D GS [4] is an efficient explicit 3D reconstruction method with differentiable 3D Gaussians as an unstructured presentation. The 3D GS at coordinate $\boldsymbol{x}$ is formulated as $G(\boldsymbol{x}) =e^{-\frac{1}{2}(\boldsymbol{x}-\boldsymbol{\mu })^T \mathbf {\Sigma }^{-1}(\boldsymbol{x}-\boldsymbol{\mu })}$, where $\mathbf {\Sigma }$ is the covariance matrix and $\boldsymbol{\mu }$ is the mean of the Gaussian primitive. $\mathbf {\Sigma }$ can be described as $\mathbf {\Sigma }=\textbf{RS}\textbf{S}^T\textbf{R}^T$ with scale $\textbf{S}$ and rotation $\textbf{R}$. Then, the 3D GS are projected onto a 2D plane with the covariance $\mathbf {\Sigma }^\prime =\mathbf {JW\Sigma }\textbf{W}^T\textbf{J}^T$, where $\textbf{W}$ is the view transformation and $\textbf{J}$ is the projective transformation’s Jacobian. The final rendered color *C* of pixel *p* is formulated as

7 $$\begin{aligned} C(p) = \sum _{i\in N}c_ia_i\prod _{j=1}^{i-1}(1-a_i), \end{aligned}$$

where *i* denotes the index of *N* 3D Gaussians. $c_i$ and $a_i$ are the Gaussian color defined by spherical harmonics and the final opacity value. *j* represents the Gaussians along the ray closer to the camera than *i*.

The proposed Endo-PairGS adopts Endo-4DGS [8] as the baseline model, with an aligned point cloud pair to initialize the 3D Gaussians. As illustrated in Fig. 2, the mean μ, scale $\textbf{S}$, and spherical harmonics $\textbf{SH}$ are initialized with an aligned point cloud pair. Additionally, the rotation $\textbf{R}$ and opacity *o* are randomly initialized. Time *t* is the conditional parameter to adjust the 3D Gaussians through an optimization network composed of HexPlane [23] and MLPs. Then, the 3D Gaussians are splatted on the 2D plane and rendered to RGB images. The 3D Gaussians initialized with our method are the density representation with a well-reconstructed geometric shape. Original pseudo-depth initialization methods lack scale information, which leads to suboptimal results.

### Loss function

Following the setting of Splatt3R [19], we adopted mean-squared error (MSE) loss and perceptual similarity to fine-tune the Splatt3R. The total reconstruction loss calculated between target $\textbf{I}_t$ and rendered output $\textbf{I}_o$ is formulated as

8 $$\begin{aligned} \mathcal {L}&= \lambda _{MSE}\mathcal {L}_{MSE}(\textbf{I}_t\odot \textbf{M}_{mix}^t, \textbf{I}_o\odot \textbf{M}_{mix}^t) \nonumber \\&+\lambda _{LPIPS}\mathcal {L}_{LPIPS}(\textbf{I}_t\odot \textbf{M}_{mix}^t, \textbf{I}_o\odot \textbf{M}_{mix}^t), \end{aligned}$$

where $\lambda _{MSE}$ and $\lambda _{LPIPS}$ are the weights of $\mathcal {L}_{MSE}$ and $\mathcal {L}_{LPIPS}$.

In dynamic scene reconstruction, pixel-wise loss, confidence-guided loss [8], and total variation (TV) loss [4] are used to train the 3D Gaussians for dynamic scene reconstruction. Pixel-wise loss used $L_1$ loss to calculate with target frame $\textbf{F}_t$, target pseudo-depth $\textbf{D}_t$, rendered frame $\textbf{F}_o$, and estimated depth $\textbf{D}_o$ as

9 $$\begin{aligned} \mathcal {L}_{pixel} = L_1(\textbf{F}_t, \textbf{F}_o)+L_1(\widehat{\textbf{D}}_t, \widehat{\textbf{D}}_o), \end{aligned}$$

where $\widehat{\textbf{D}}_t, \widehat{\textbf{D}}_o$ are $\textbf{D}_t,\textbf{D}_o$ normalized to (0, 1). Confidence-guided loss is calculated with the generated confidence map $\textbf{W}$ as weights, which is computed as

10 $$\begin{aligned} \mathcal {L}_{con}&= \lambda _f\mathbb {E}[\frac{1}{2}\Vert \textbf{F}_t-\textbf{F}_o\Vert _2\oslash \textbf{W}^2+\textrm{log}(\textbf{W})] \nonumber \\&+\lambda _d\mathbb {E}[\frac{1}{2}\Vert \widehat{\textbf{D}}_t-\widehat{\textbf{D}}_o\Vert _2\oslash \textbf{W}^2+\textrm{log}(\textbf{W})], \end{aligned}$$

where $\mathbb {E}(\cdot )$ is the expectation, and $\textrm{log}(\cdot )$ added as a regularization term. ⊘ is the element-wise division. $\lambda _f, \lambda _d$ are the weights of the frame part and the depth part. Total loss of step 2 is calculated as $\mathcal {L}_{gs} = \mathcal {L}_{pixel}+\mathcal {L}_{con}+\mathcal {L}_{TV}$.

**Table 2:** Quantitative comparison on EndoNeRF and StereoMIS datasets. The tiny numbers are standard deviations under each metric. The best and second-best results are written in bold and underlined

	EndoNeRF-Cutting			EndoNeRF-Pulling			StereoMIS (P1)		
Methods	PSNR↑	SSIM↑	LPIPS↓	PSNR↑	SSIM↑	LPIPS↓	PSNR↑	SSIM↑	LPIPS↓
EndoNeRF [5] (MICCAI ’22)	35.71	0.933	0.059	36.50	0.941	0.054	31.12	0.846	0.148
	±1.02	±0.008	±0.007	±2.09	±0.011	±0.015	±0.74	±0.011	±0.010
EndoSurf [6] (MICCAI ’23)	36.55	0.953	0.055	37.21	0.955	0.050	31.29	0.865	0.168
	±1.22	±0.006	±0.006	±2.50	±0.010	±0.014	±0.76	±0.009	±0.013
ForPlane-32k [15] (TMI ’24)	36.85	0.944	0.045	37.82	0.955	0.042	32.19	0.843	**0**.**143**
	±1.44	±0.012	±0.010	±2.78	±0.017	±0.017	±1.48	±0.025	±0.035
Endo-4DGS [8] (MICCAI ’24)	36.56	0.955	0.032	37.85	0.959	0.043	32.17	0.850	0.192
	±2.59	±0.019	±0.008	±3.79	±0.020	±0.025	±3.03	±0.045	±0.094
EndoGaussian [13] (TMI ’25)	38.38	0.963	0.030	37.09	0.956	0.039	32.79	0.858	0.182
	±2.43	±0.014	±0.008	±3.63	±0.017	±0.021	±2.10	±0.030	±0.040
Deform3DGS [10] (MICCAI ’24)	38.72	0.966	0.030	38.33	0.960	0.040	33.35	**0**.**871**	0.169
	±2.15	±0.010	±0.008	±3.28	±0.017	±0.019	±2.08	±0.029	±0.043
SurgicalGS [14] (MICCAI ’25)	**38**.**85**	**0**.**967**	0.030	38.03	0.959	0.039	32.98	0.867	0.172
	±2.14	±0.009	±0.008	±3.00	±0.018	±0.019	±2.08	±0.031	±0.043
Endo-PairGS (Ours)	38.33	**0**.**967**	**0**.**020**	**38**.**96**	**0**.**967**	**0**.**022**	**33**.**56**	**0**.**871**	0.146
	±3.15	±0.021	±0.007	±4.30	±0.019	±0.014	±2.85	±0.042	±0.052

**Table 3:** Quantitative comparison on the StereoMIS dataset with more challenging situations. The tiny numbers are standard deviations under each metric. The best and second-best results are written in bold and underlined

	StereoMIS (P2-2)			StereoMIS (P2-5)			StereoMIS (P3)		
Methods	PSNR↑	SSIM↑	LPIPS↓	PSNR↑	SSIM↑	LPIPS↓	PSNR↑	SSIM↑	LPIPS↓
EndoNeRF [5] (MICCAI ’22)	28.91	0.703	0.261	26.49	0.688	0.274	29.85	0.751	0.209
	±0.78	±0.027	±0.038	±0.82	±0.012	±0.008	±0.30	±0.011	±0.009
EndoSurf [6] (MICCAI ’23)	30.01	0.780	0.198	26.51	0.738	0.277	30.46	0.811	0.188
	±1.35	±0.043	±0.046	±0.84	±0.015	±0.010	±0.36	±0.009	±0.008
ForPlane-32k [15] (TMI ’24)	28.01	0.659	0.286	28.48	0.759	0.170	31.10	0.801	0.139
	±1.09	±0.047	±0.065	±1.48	±0.061	±0.061	±1.15	±0.041	±0.034
Endo-4DGS [8] (MICCAI ’24)	31.45	0.812	0.141	29.06	0.817	0.152	31.21	0.831	0.155
	±2.80	±0.098	±0.080	±1.12	±0.035	±0.035	±0.67	±0.021	±0.017
EndoGaussian [13] (TMI ’25)	29.82	0.759	0.169	28.28	0.782	0.173	31.26	0.821	0.166
	±2.48	±0.091	±0.065	±1.15	±0.043	±0.031	±0.76	±0.019	±0.013
Deform3DGS [10] (MICCAI ’24)	30.69	0.789	0.144	29.16	0.813	0.147	31.70	0.834	0.160
	±2.69	±0.092	±0.063	±1.27	±0.042	±0.033	±0.65	±0.017	±0.013
SurgicalGS [14] (MICCAI ’25)	30.19	0.775	0.178	29.07	0.805	0.168	31.97	0.841	0.155
	±2.00	±0.069	±0.054	±1.03	±0.034	±0.025	±0.57	±0.016	±0.011
Endo-PairGS (Ours)	**32**.**16**	**0**.**833**	**0**.**100**	**29**.**75**	**0**.**846**	**0**.**103**	**32**.**47**	**0**.**871**	**0**.**110**
	±2.99	±0.093	±0.050	±1.37	±0.034	±0.023	±0.86	±0.021	±0.015

**Fig. 3:**
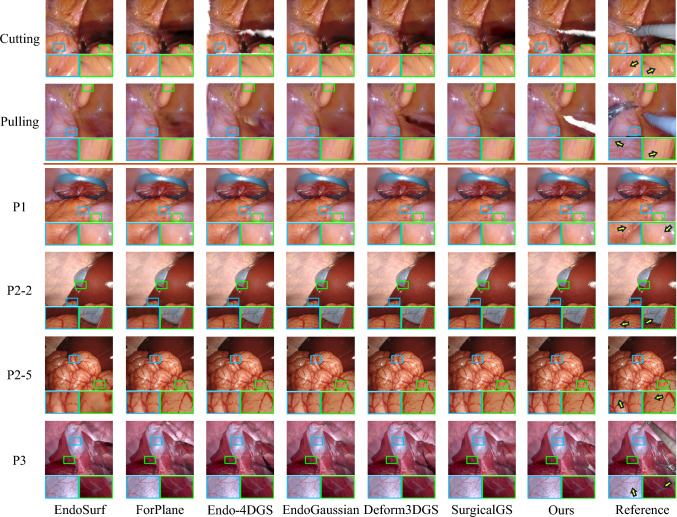
The visualized comparison of six dynamic scenes in EndoNeRF and StereoMIS datasets. We zoom in on the details for better evaluation

**Fig. 4:**
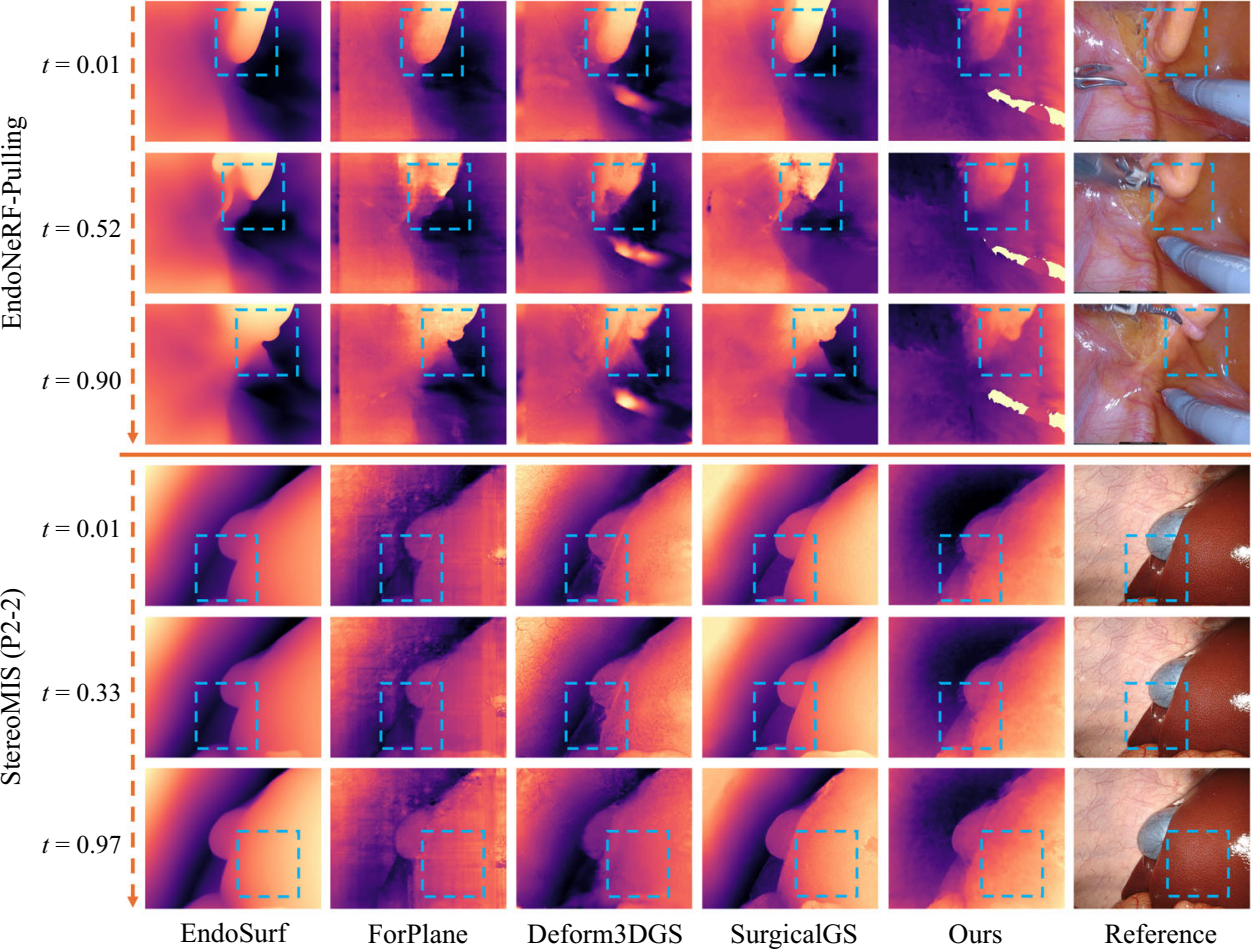
The visualized comparison of the rendered depth in the EndoNeRF-Pulling and StereoMIS (P2-2) datasets. t∈[0,1] denotes the normalized frame timestamp (0 = first frame, 1 = last frame)

## Experiments

### Experimental settings

**Dataset and evaluation.** To fully exhibit the performance compared to previous SOTA methods, we collected 20 dynamic endoscopic scenes with 2,714 frames from the EndoNeRF [5] and the StereoMIS [20] datasets. The EndoNeRF dataset contains six stereo endoscopic video clips extracted from Da Vinci robotic prostatectomy procedures, and the StereoMIS dataset consists of stereo endoscopic videos from in vivo porcine tissues, including challenging scenes with breathing motions, tissue deformations, and resections. Two of the collected scenarios are from the EndoNeRF dataset, and the other 18 sequences are extracted from the StereoMIS dataset. Following Endo-4DGS [8], we split the train and test sets with the 7:1 strategy with a resolution of 640×512 pixels. Splatt3R is fine-tuned on the full training datasets, and six scenes with different tool occlusion and tissue deformation situations were chosen for the evaluation of dynamic reconstruction, as detailed in Table 1. We evaluate reconstruction quality with PSNR, SSIM, and LPIPS as metrics. All the compared methods were retrained to maintain fairness.

**Implementation details.** Splatt3R was fine-tuned on all 20 dynamic scenes for 7500 iterations with a learning rate of $5\times 10^{-6}$ and a batch size of 16. The weights of $\lambda _{MSE}$ and $\lambda _{LPIPS}$ are set to 1.0 and 0.25 for optimization. In the optical flow-based mask, the mask threshold $\lambda _{thr} = 0.2$, and the mask weight α=0.5. Our Endo-PairGS was initialized with an aligned point cloud pair from frames (1, 31) and trained for 1,000 iterations in the coarse stage and 10,000 iterations in the fine stage with an initial learning rate of $1.6\times 10^{-3}$. Other settings followed Endo-4DGS [8]. All the experiments were conducted with the PyTorch framework on the NVIDIA A100 GPU. The fine-tuning of Splatt3R cost about 7 h, and the training of dynamic 3D reconstruction on one scene needed around 10 min. When initializing with paired or multiple point clouds, we leveraged the Open3D library to remove duplicated and statistical outlier points for overlap information.

### Comparison results

We evaluated our method on six dynamic scenes with the comparison of previous SOTA NeRF and GS-based methods, including EndoNeRF [5], EndoSurf [6], ForPlane [15], Endo-4DGS [8], EndoGaussian [13], Deform3DGS [10], and SurgicalGS [14].

#### Quantitative analysis

Table 2 shows the comparison of three dynamic scenes with an almost fixed camera. Our Endo-PairGS achieved the best results on EndoNeRF-Pulling and comparable performance on the other two scenes, especially for LPIPS scores. It is necessary to declare that EndoGaussian, Defrom3DGS, and SurgicalGS are trained with ground-truth depth on the EndoNeRF (both cutting and pulling) dataset for their outstanding performance. To further demonstrate the performance of our method, the experiment on three more challenging scenes is shown in Table 3. The proposed Endo-PairGS exhibited outstanding improvements on all metrics when facing scenarios with greater camera movement and tissue deformation. In addition to achieving superior reconstruction results, our method maintained the computational efficiency of Endo-4DGS in dynamic reconstruction, utilizing 4 gigabytes of memory during training and achieving an inference speed of 100 FPS.

#### Qualitative comparison

In Fig. 3, the visualized results on six dynamic scenes demonstrate our performance in texture reconstruction for the smooth surface. Compared to the render results of P2-2 and P2-5, our method produced more details and sharper edges of the blood vessels on the intestine surface, which were closer to references. The proposed approach also showed greater clarity and less blur in the details of the reflective surfaces on cutting and P1 when exposed to bright light. We further performed a visual comparison of depth to demonstrate the geometric structure. Figure 4 illustrates the predicted depth of rendered frames of different timestamps in the EndoNeRF-Pulling and StereoMIS (P2-2) datasets. Although our result was quite different from others using ground-truth depth as guidance in EndoNeRF-Pulling, it exhibited a sharp edge and smooth surface in the rectangular deformed region. When using pseudo-depth for initialization and guidance in StereoMIS (P2-2), our performance in the rectangular area exhibited more geometric details and smoother depth distribution.

**Table 4:** Ablation study of different training strategies and settings on the EndoNeRF and StereoMIS datasets. PCD refers to point cloud. The best results are written in bold

	EndoNeRF-Pulling			StereoMIS (P2-2)		
Methods	PSNR↑	SSIM↑	LPIPS↓	PSNR↑	SSIM↑	LPIPS↓
w/o fine-tuning	38.51±4.34	0.965±0.020	0.027±0.015	31.90±2.84	0.832±0.089	0.106±0.054
Init. w/ 1 PCD	38.47±4.12	0.964±0.020	0.029±0.017	32.11±2.83	**0**.**836**±0.085	0.110±0.055
Init. w/ 3 PCDs	**38**.**98**±4.43	**0**.**967**±0.021	**0**.**020**±0.013	32.14±3.13	0.834±0.094	**0**.**089**±0.046
Init. w/ 50% of 3 PCDs	38.96±4.13	**0**.**967**±0.019	0.023±0.013	**32**.**16**±3.04	0.835±0.092	0.098±0.050
Init. w/ paired PCDs	38.96±4.30	**0**.**967**±0.019	0.022±0.014	**32**.**16**±2.99	0.833±0.093	0.100±0.050
Init. w/ interval 10	38.90±4.26	0.966±0.021	0.025±0.015	32.09±3.09	0.834±0.092	0.099±0.050
Init. w/ interval 20	**38**.**99**±4.29	**0**.**967**±0.020	0.023±0.014	32.13±3.10	0.835±0.091	**0**.**098**±0.049
Init. w/ interval 30	38.96±4.30	**0**.**967**±0.019	0.022±0.014	32.16±2.99	0.833±0.093	0.100±0.050
Init. w/ interval 40	38.81±4.60	0.966±0.022	**0**.**021**±0.013	**32**.**17**±2.96	**0**.**836**±0.091	0.100±0.051
Init. w/ $\lambda _{thr}=0.2$	**38**.**88**±4.30	**0**.**967**±0.020	**0**.**022**±0.014	**32**.**16**±3.02	**0**.**834**±0.091	0.098±0.049
Init. w/ $\lambda _{thr}=0.4$	38.77±4.51	**0**.**967**±0.020	**0**.**022**±0.014	32.09±3.07	**0**.**834**±0.093	**0**.**097**±0.049
Init. w/ $\lambda _{thr}=0.6$	38.87±4.26	**0**.**967**±0.020	**0**.**022**±0.014	**32**.**16**±3.04	0.833±0.093	0.099±0.050

### Ablation study

We conducted the ablation study with different settings, including fine-tuning, initialization strategy, and hyperparameters, shown in Table 4. To demonstrate the effectiveness of our fine-tuning strategy on Splatt3R, we initialized the 3D Gaussians with an aligned point cloud pair generated directly by pre-trained Splatt3R. The results shown in the first row are degraded on all three metrics compared to the one using fine-tuned Splatt3R, mainly due to the domain gap that causes a predicted error in the camera’s intrinsics. The visualized fine-tuning results are shown in the Supplementary Material.

To determine the optimized quantities of point clouds for initialization, we retrained the Endo-PairGS with a point cloud from a single frame and aligned triple point clouds from frames (1, 31, 61), respectively. The results from a single point cloud are inferior to those that use paired or triple point clouds in the scene with large surgical tool occlusion, e.g., EndoNeRF-Pulling. However, the results on the non-surgical tool scene, e.g., StereoMIS (P2-2), show almost the same performance across the three initialization methods. The results of triple point clouds almost achieved the best performance, with the benefit of more valid information. More 3D Gaussians will introduce more computation during training, so we randomly sampled 50% of points from the aligned point cloud of 3 frames to reduce the training cost. Using the same magnitude of initialized 3D Gaussians, the models nearly achieved identical performance when initialized with either paired or triple point clouds.

The ablation of the frame pair interval and the fine-tuning mask threshold are listed in the last two groups of Table 4. We choose four different frame pair intervals of 10, 20, 30, and 40. With the results on two scenes, there are no obvious variances in performance. Commonly, the interval of frame pairs larger than 10 is enough, but it depends on the tool and camera movements in different video sequences. We set three thresholds, 0.2, 0.4, and 0.6, to define the overlap region between input pairs. There are almost the same results as exhibited. It demonstrates that the threshold of 0.2 already contained the most overlap region, and the complementary random mask between input pairs is more crucial to maintaining the gradient in the overlap region.

## Discussion

Based on the results from six endoscopic scenes, our proposed method showed greater improvements in more challenging scenarios. The aligned point cloud pairs will contain additional information that highlights the differences between the two frames, allowing for better performance in sequences with significant camera movements, such as colonoscopic videos. Some other methods [10] utilized depth and camera extrinsics from multiple frames to initialize the 3D Gaussians. However, the unscaled predicted depths will be misaligned in the generated point cloud, resulting in a discontinuous surface. Due to data and label limitations, we only fine-tuned the Gaussian head of Splatt3R with 2D loss functions, restricting 3D reconstruction performance. Limited to the unlabeled datasets, the quantitative results primarily focused on rendered 2D views. It is necessary to collect more annotated datasets with 3D ground truth, such as simulated data or stereo data, to improve the 3D geometric performance of fine-tuned Splatt3R and evaluate it with 3D metrics like Chamfer distance.

Some boundary conditions, like rapid camera movements or extreme topological changes, will yield inadequate optical flow estimation results for RAFT. To ensure the accuracy of optical flow-based masks, it is crucial to leverage a beneficial selection strategy for input frames to avoid large content differences. Commonly, it is efficient to ensure a valid overlapping region between input frames by using shorter video sequences or limiting the input frame interval, which may improve the estimation accuracy of optical flow. An uncertainty-aware flow estimation is also a beneficial choice for scenarios with surgical resections and severe tissue tearing.

## Conclusion

In this study, we proposed an efficient initialization method for Gaussian splatting with aligned point cloud pairs in dynamic endoscopic scene reconstruction. To do so, we fine-tuned Splatt3R on dynamic endoscopic scenarios with the optical flow-based mask, which aims to generate a well 3D-reconstructed and aligned point cloud pair. Then, the pseudo-point cloud pair and corresponding depth are used to initialize and train a 4D GS model for dynamic reconstruction. The demonstrated results show that aligned point cloud pairs contain more complete tissue information and embedded camera intrinsics, which lead to superior initialization performance. We fine-tuned Splatt3R on endoscopic data, but the limited data restrict its performance. In future work, we will collect more high-quality labeled endoscopic data to improve the alignment accuracy of deformed frame pairs.

## Data Availability

The code and split of data will be released publicly on GitHub once this paper is accepted.
